# Cancer Patient Tissueoid with Self‐Homing Nano‐Targeting of Metabolic Inhibitor

**DOI:** 10.1002/advs.202105264

**Published:** 2021-12-22

**Authors:** Hyo‐Jin Yoon, Young Shin Chung, Yong Jae Lee, Seung Eun Yu, Sewoom Baek, Hye‐Seon Kim, Sang Wun Kim, Jung‐Yun Lee, Sunghoon Kim, Hak‐Joon Sung

*Adv. Sci*. **2021**, *8*, 2102640

https://doi.org/10.1002/advs.202102640

In the originally published article, one grant number was missing in the acknowledgements. Please find the correct acknowledgements here:

This work was financially supported by kNRF 2019R1A2C22010802. The metabolic inhibitor was provided by HaimBio LTD, Seoul, Korea. The authors acknowledge the doctors and nurses of Obstetrics and Gynaecology in Severance hospital (Seoul, Korea), who helped provide clinical specimens. This work was also supported by the Korea Medical Device Development Fund grant funded by the Korea government (the Ministry of Science and ICT, the Ministry of Trade, Industry and Energy, the Ministry of Health & Welfare, the Ministry of Food and Drug Safety) (Project Number: 1711138302, KMDF_PR_20200901_0152).

